# The influence of dietary supplementation with ginger ethanol extract on laying hens’ production performance, antioxidant capacity, and gut microbiota

**DOI:** 10.3389/fvets.2025.1652982

**Published:** 2025-09-23

**Authors:** Sanjun Jin, Jiajia Shi, Mixue Zhao, Xinhe Liu, Kaige Yang, Enci Shang, Ping Wang, Chaoqi Liu, Lijun Wang, Xinxin Li, Qingqiang Yin, Zhiguang Yue, Xiaowei Dang, Juan Chang

**Affiliations:** ^1^College of Animal Science and Technology, Henan Agricultural University, Zhengzhou, China; ^2^Henan Anjin Biotechnology Co., Ltd., Xinxiang, China; ^3^Henan Delin Biological Product Co. Ltd., Xinxiang, China

**Keywords:** ginger ethanol extract, laying hens, egg quality, antioxidation, gut microbiota

## Abstract

This study aimed to investigate the effects of ginger ethanol extract (GEE) on the production performance, egg quality, serum biochemistry, antioxidant capacity, and gut microbiota of Dawu Golden Phoenix laying hens. The study included 288 Dawu Golden Phoenix laying hens, aged 44 weeks, which were randomly divided into four groups: CON (basal diet), GEE 200 (basal diet + 200 mg kg^−1^ GEE), GEE 400 (basal diet + 400 mg kg^−1^ GEE), and GEE 600 (basal diet + 600 mg kg^−1^ GEE). The results demonstrated that dietary GEE significantly increased apparent ether extract (EE) digestibility (*p* < 0.05) compared to the basal diet. Hens that were fed GEE diets exhibited an improved feed-to-egg ratio (FCR) and increased levels of serum total protein (TP) and high-density lipoprotein cholesterol (HDL-C) (*p* < 0.05), along with reduced levels of serum total triglycerides (TG) and low-density lipoprotein cholesterol (LDL-C) (*p* < 0.05). Furthermore, dietary GEE (600 mg kg^−1^) significantly increased serum antioxidant capacity and estradiol (E_2_) levels (*p* < 0.05). No significant differences were observed in alpha and beta diversity across the groups, except for the Chao index (*p* < 0.05). *Bacteroidota* and *Firmicutes* predominated at the phylum level, while *Bacteroides* emerged as the dominant genus. The *Firmicutes*-to-*Bacteroidota* ratio tended to increase in the GEE400 and GEE600 groups. At the genus level, hens that were fed 600 mg kg^−1^ of GEE showed significantly higher abundances of *Faecalibacterium* and *Rikenellaceae_RC9_gut_group*, but lower abundances of *Bacteroides* and *unclassified_o_Bacteroidales* compared to the CON group (*p* < 0.05). Correlation analysis revealed that *Lactobacillus* and *Faecalibacterium* were positively correlated with antioxidant capacity, indicating that GEE improved antioxidant status by increasing the relative abundances of beneficial intestinal probiotics. The paper concludes with a discussion that GEE supplementation improved animal production by reducing the FCR value and enhancing apparent EE digestibility, while modulating serum biochemical parameters. It also enhanced the antioxidant function by regulating gut microbiota. Therefore, the optimal addition of GEE as a feed additive for laying hens is 600 mg kg^−1^.

## 1 Introduction

With increasing concerns about food safety, natural feed additives have gained widespread application in laying hen diets due to their eco-friendly characteristics and multifaceted bioactive properties (1). With increasing concerns about food safety, natural feed additives have gained widespread application in laying hen diets due to their eco-friendly characteristics and multifaceted bioactive properties. They effectively improve growth performance and feed conversion ratio (2), a egg quality, antioxidant status, and intestinal health (3, 4), thereby enhancing overall health parameters. Furthermore, phytogenic compounds exhibit the potential to optimize health parameters in poultry production systems. Consequently, the strategic incorporation of natural feed additives into poultry diets has emerged as a focal point for contemporary scientific research.

*Ginger ethanol extract* (GEE), one of the most prevalent natural feed additives, is derived from *Zingiber officinale* rhizomes and exhibits potent antioxidant, anti-inflammatory, and antimicrobial properties, along with modulatory effects on neurological activity (5–7). Pretreatment with GEE at doses of 100 and 500 mg kg^−1^ body weight (oral administration) mitigates radiation-induced intestinal oxidative stress in mice (8). Growing evidence indicates that ginger-derived compounds, including GEE, enhance growth performance, egg quality, and antioxidant status in poultry (9, 10). Collectively, these findings demonstrate that ginger supplementation improves nutrient absorption, growth efficiency, and oxidative homeostasis in livestock. Recent research highlights a significant correlation between antioxidant capacity, gut microbiota composition, and production performance in animals (11). Despite these advances, the mechanistic effects of GEE on laying hen physiology—particularly regarding egg production, antioxidant pathways, and microbiome–host crosstalk—remain underexplored.

Therefore, the primary objective of this study is to evaluate the effects of dietary ginger ethanol extract (GEE) supplementation on production performance, egg quality, serum antioxidant biomarkers, and gut microbiota composition in Dawu Golden Phoenix laying hens during the production period. Furthermore, this study aims to determine the optimal GEE dosage for practical application as a natural feed additive.

## 2 Materials and methods

The experimental protocols were approved by the Institutional Animal Care and Use Committee (IACUC) of Henan Agricultural University (Approval No. 11-0099-2023), in compliance with the Guidelines for the Ethical Treatment of Laboratory Animals.

### 2.1 Materials

GEE was generously donated by Henan Anjin Biotechnology Co., Ltd. (Henan, China). Ginger rhizomes were thoroughly rinsed to remove soil and surface impurities. Subsequently, the cleaned samples were precisely sliced into uniform pieces approximately 3 mm in thickness to ensure consistent drying and extraction performance. These slices were then subjected to hot-air drying in an electric thermostatic drying oven at a temperature of 50 °C for 48 h, effectively reducing the moisture content while preserving thermolabile bioactive compounds. Thereafter, the dried slices were ground using a grinder and sieved through a 60-mesh (0.28 mm) sieve. Ginger essential oils were extracted via ethanol as follows: dried ginger powder was mixed with 75% ethanol at a liquid-to-solid ratio of 8 mL g^−1^, extracted at 20 °C–60 °C for 1 h, and then filtered. The resulting essential oil was collected and stored at 4 °C in the dark, preventing its exposure to direct light. The process and efficacy of GEE are as detailed in the patent.^1^ A powdered additive was prepared by mixing SiO₂ as a carrier with GEE at a ratio of 2:1, followed by storage in a sealed container in a cool and dry place. The primary compounds of ginger essential oils were analyzed by Suzhou Panomix Biomedical Tech Co. Ltd. (Suzhou, China) using ultrahigh performance liquid chromatography–tandem mass spectrometry (UPLC–MS/MS) (Supplementary Table S1). The major compounds in GEE, including 6-gingerol, 8-gingerol, and 10-gingerol, were quantified using high-performance liquid chromatography coupled with tandem mass spectrometry (HPLC–MS/MS) (Supplementary Table S2).

### 2.2 Birds, experiment design, and management

In this study, GEE was added to the diet of growing Japanese quail at concentrations of 200, 400, and 600 mg kg^−1^, based on the findings of Mohamed et al. (12), which demonstrated that the body weight (BW) of chicks received 250 and 500 mg kg^−1^ ginger powder significantly increased at 5 and 6 weeks of age, respectively, and Dosoky et al. (13) for laying Japanese quail, which demonstrated that ginger powder (250 and 500 mg kg^−1^) enhanced blood serum properties and improved reproductive and productive performance. A total of 288 healthy, 44-week-old Dawu Golden Phoenix laying hens were obtained from the Poultry Breeding Center of Henan Province (Tongxu, China) and used in a 56-day study, following a 7-day acclimation period. Initial egg production rates showed no significant intergroup differences. Laying hens were randomly allocated into four dietary treatment groups (*n* = 6 replicates/group, 12 hens/replicate): CON (basal diet), GEE 200 (basal diet + 200 mg kg^−1^ GEE), GEE 400 (basal diet + 400 mg kg^−1^ GEE), and GEE 600 (basal diet + 600 mg kg^−1^ GEE). Laying hens were housed in a metal cage (L × W × H = 38.5 × 38 × 34 cm), with three hens per cage. Four cages constituted one replicate unit under a 16: 8 h light: dark photoperiod and at 25 °C–28 °C. Laying hens were provided with ad-libitum constant access to powdered diets and water. The basal diets were formulated according to the National Research Council (NRC) (Table 1) (14) and the Chinese chicken feeding standards (NY/T 823-2020-Performance Terminology and Measurements for Poultry). To ensure uniform mixing in diets, GEE-containing feed was made by mixing GEE with SiO_2_ as solid support. At the end of the trial, six laying hens per replicate group were humanely euthanized by cervical dislocation. Samples were collected within 3 min of euthanasia, following the American Veterinary Medical Association (AVMA) Guidelines for the Euthanasia of Animals (2020).

**Table 1:** Ingredient and nutrient composition of experimental diets (air-dry basis).

Ingredients	Content, %	Nutrient levels	Content
Corn	58.62	Metabolic energy^b^, KJ kg^−1^	11.39
Wheat	3.63	Crude protein^c^, %	15.29
Straw meal	1.00	Crude fat^c^, %	2.27
Soybean meal	24.08	Calcium^c^, %	3.49
Soybean oil	1.00	Total phosphorus^c^, %	0.58
CaCO_3_	8.80	Lysine^b^, %	0.81
DCP	1.40	Methionine^b^, %	0.36
Salt	0.37	Metabolic energy^b^, KJ kg^−1^	11.39
Methionine	0.10		
Premix^a^	1.00		
Total	100.00		

a The premix provides a per kg ration of the following elements: copper (20 mg), iron (80 mg), manganese (80 mg), zinc (59 mg), selenium (0.12 mg), iodine (0.55 mg), choline (250 mg), vitamin A (60,100 IU), and vitamin D3. The premix provides the following per kg ration: 20,000 IU of vitamin E, 200 mg of vitamin K3, 20 mg of vitamin B1, 60 mg of vitamin B2, 30 mg of niacin, 10 mg of pantothenic acid, and 0.5 mg of folic acid, in addition to 0.22 mg of biotin. The concentration of pantothenic acid is 10 mg, folic acid is 0.5 mg, and biotin is 0.22 mg.

b The metabolizable energy, lysine, and methionine values were calculated.

c Values were measured.

### 2.3 Productive performance and sample collection

Egg weight and production were recorded daily, while feed intake was measured weekly. These data were used to calculate average daily feed intake (ADFI), laying rate, average egg weight, average daily egg weight (ADEW), and feed-to-egg ratio (FCR = total feed intake/total egg weight).

Fecal samples were collected over a continuous 72-h period (days 53–55) using the total collection method for assessing nutrient digestibility. Proximate analyses of crude protein (CP; GB/T 6432-2018), dry matter (DM; GB/T 6435-2014), ether extract (EE; GB/T 6433-2006), phosphorus (GB/T 6437-2018), and calcium (GB/T 6436-2018) were performed according to Chinese national standards. Apparent nutrient digestibility was determined using acid-insoluble ash (AIA) as an inert marker, and it is calculated as follows:


$$\begin{array}{l} \text{Apparent digestibility of nutrients}(\%)=\\ 1-\frac{\text{content of nutrients in feces}}{\text{content of nutrients in feed}}\times \frac{\text{indicator content in feed}}{\text{indicator content in feces}} \end{array}$$

Blood samples were collected from laying hens via wing vein venipuncture into ethylenediaminetetraacetic acid (EDTA)-coated vacuum tubes (BD Biosciences) following more than 8 h of overnight fasting. The samples were centrifuged at 1000 × *g* for 5 min (4 °C). After separation, the serum was immediately stored at −80 °C until analysis. Concurrently, during euthanasia, cecal contents were aseptically collected into cryovials, snap-frozen in liquid nitrogen, and stored at −80 °C for 16S rRNA sequencing.

### 2.4 Quality assessment of eggs

At the 47th and 51st weeks of hen age (corresponding to 4- and 8-week experimental intervals), 36 eggs per treatment group were randomly selected. The eggs were stored at 25 ± 0.5 °C under 60% relative humidity and analyzed within 24 h post-collection. The egg shape index was evaluated by measuring the length and width using a digital Vernier caliper (606-01, Harbin Tools Electrical Co., Ltd., Harbin, China; accuracy, 0.2 mm). Eggshell thickness (ESS) was determined using a digital peacock dial gauge (P-1 Model, Meg Co. Ltd., Ozaki, Japan), after the removal of shell membrane. Haugh units, albumen height, and yolk color were recorded using an automated egg quality analysis instrument (DET-60000, NABEL Co., Ltd., Japan).

### 2.5 Serum biochemical parameters

Serum biochemical parameters were measured using an automatic biochemistry analyzer (Hitachi No. 7600-020, Hitachi High-Technologies Corporation, Tokyo, Japan) on day 56 of the trial. This analyzer quantified alanine aminotransferase (ALT), aspartate aminotransferase (AST), total protein (TP), albumin (ALB), total cholesterol (TC), triglycerides (TG), high-density lipoprotein cholesterol (HDL-C), and low-density lipoprotein cholesterol (LDL-C).

### 2.6 Serum antioxidant capacities

On day 56 of the trial, serum concentrations of total antioxidant capacity (T-AOC), catalase (CAT), superoxide dismutase (SOD), glutathione peroxidase (GSH-Px), and malondialdehyde (MDA) were measured using antioxidant assay kits (Nanjing Jiancheng Bioengineering Co., Ltd., Nanjing, China).

### 2.7 Serum reproductive hormone content

On day 56 of the trial, serum concentrations of progesterone (P_4_), luteinizing hormone (LH), estradiol (E_2_), and follicle-stimulating hormone (FSH) were measured using ELISA assay kits (Nanjing Jiancheng Bioengineering Co., Ltd., Nanjing, China).

### 2.8 Microbial analysis

Total microbial genomic DNA was extracted from the cecal content of laying hens using the QIAamp Fast DNA Stool Mini Kit (Qiagen, Hilden, Germany). DNA purity and concentration were assessed using 1% agarose gel electrophoresis and a Nanodrop-1000 instrument (Thermo Fisher Scientific, Waltham, MA, USA), followed by dilution to 1 ng μl^−1^ with sterile water. The V3–V4 region of the bacterial 16S rRNA gene was amplified by PCR, with the amplicons verified on 2% agarose gel. PCR products were then purified using the Qiagen Gel Extraction Kit (Qiagen, Hilden, Germany) following the manufacturer’s instructions. This purification step ensured the removal of any non-specific products or impurities, thereby enhancing the quality of the amplicons for downstream analyses.

Sequencing libraries were prepared using the TruSeq DNA PCR-Free Sample Preparation Kit (Illumina, USA) following the manufacturer’s protocol. The quality of the constructed libraries was assessed using a Qubit fluorometer (ThermoScientific) to ensure accurate quantification and integrity. Subsequently, high-throughput sequencing was conducted on an Illumina MiSeq platform (Illumina) at Major Bio. This approach ensured high-quality sequencing data and robust downstream analyses for the study.

Initial tags were filtered to ensure cleanliness and quality, while chimeric sequences were removed. Subsequently, the sequences with a similarity of 97% were grouped into operational taxonomic units (OTUs) using QIIME2 software. Alpha diversity indices, including Chao1, Shannon, and Simpson, and beta diversity were calculated using QIIME2 software and displayed with R software. Beta diversity was calculated using the Bray–Curtis index and visualized through principal coordinate analysis (PCoA) and non-metric multidimensional scaling (NMDS) plots. To infer functional potential, we performed Reconstruction of Unobserved States 2 (PICRUSt2) analysis of on the normalized amplicon sequence variant (ASV) table. This process predicted the abundance of Kyoto Encyclopedia of Genes and Genomes (KEGG) orthologs (KOs), which were then mapped to their respective biochemical pathways.

### 2.9 Data analysis

All data obtained in this experiment were statistically analyzed using the IBM SPSS Statistics 26.0 statistical package (SPSS Inc., Chicago, IL, USA). One-way analysis of variance (ANOVA) was used to compare the differences between GEE dietary supplements. Prior to ANOVA, the assumptions of normality (Shapiro–Wilk test) and homogeneity of variances (Levene’s test) were verified, and the data met these assumptions. The differences between the groups were assessed by Duncan’s multiple comparisons. To evaluate the dose–response trends, polynomial contrasts were used to test for linear and quadratic effects of dietary GEE supplementation. All results are presented as means ± standard deviation (mean ± SD), with statistical significance set at a *p*-value of <0.05.

## 3 Results

### 3.1 Production performance of laying hens among different GEE treatments

Effects of GEE on the production performance of laying hens are presented in Table 2. No significant differences were observed in the ADFI, ADEW, and laying rate among the four treatment groups during the trial period. However, the FCR of laying hens was significantly decreased with GEE supplementation during weeks 48–51 and displayed a linear dose response (*p* = 0.034), with the lowest value observed in the 600 mg kg^−1^ GEE group. This demonstrates that prolonged GEE supplementation beneficially affected the feed efficiency. No significant differences in the FCR were detected during weeks 44–47 or over the entire trial period (weeks 44–51).

**Table 2:** Effects of ginger ethanol extract on the production performance in laying hens.

Items	GEE, mg kg^−1^				*p*-Value	*p*-Value	
	CON	200	400	600		Linear	Quadratic
ADFI, g							
44–47 weeks	109.42 ± 5.36	107.61 ± 5.34	105.57 ± 3.99	105.59 ± 3.15	0.593	0.177	0.382
48–51 weeks	112.97 ± 3.11	109.79 ± 8.54	107.92 ± 0.70	107.25 ± 4.70	0.427	0.098	0.235
44–51 weeks	111.20 ± 4.48	108.70 ± 6.69	106.74 ± 2.94	106.51 ± 3.81	0.178	0.052	0.083
ADEW, g							
44–47 weeks	52.84 ± 1.50	52.42 ± 1.37	51.47 ± 1.55	52.03 ± 1.57	0.453	0.220	0.347
48–51 weeks	53.52 ± 0.32	52.5 ± 0.62	51.63 ± 1.91	52.69 ± 2.03	0.118	0.228	0.109
44–51 weeks	53.18 ± 0.88	52.46 ± 0.67	51.54 ± 1.62	52.36 ± 1.72	0.226	0.176	0.086
Laying rate, %							
44–47 weeks	93.15 ± 2.98	92.66 ± 2.73	92.81 ± 3.11	92.86 ± 2.98	0.994	0.908	0.809
48–51 weeks	91.93 ± 1.19	91.27 ± 1.67	91.32 ± 3.44	92.31 ± 4.16	0.914	0.796	0.756
44–51 weeks	92.54 ± 1.98	91.96 ± 1.72	92.06 ± 3.31	92.58 ± 3.47	0.970	0.937	0.883
FCR							
44–47 weeks	2.07 ± 0.06	2.05 ± 0.06	2.05 ± 0.06	2.03 ± 0.06	0.711	0.250	0.521
48–51 weeks	2.11 ± 0.01^a^	2.10 ± 0.03^ab^	2.09 ± 0.08^ab^	2.04 ± 0.07^b^	0.150	0.034	0.080
44–51 weeks	2.09 ± 0.03	2.08 ± 0.03	2.08 ± 0.06	2.03 ± 0.07	0.259	0.064	0.156

^a, b^Means in the same row with different superscripts differ significantly (*p* < 0.05).

ADFI, average daily feed intake; ADWE, average daily weight eggs; FCR, feed egg ratio: total feed intake/total egg weight.

### 3.2 Apparent nutrient digestibility of laying hens among different GEE treatments

The apparent nutrient digestibility of laying hens is shown in Table 3. No significant differences were recorded among groups regarding the apparent nutrient digestibility of DM, CP, Ca, and P. However, the addition of GEE significantly increased the apparent EE digestibility in laying hens compared to the control group (*p* < 0.05).

**Table 3:** Effects of ginger ethanol extract on nutrient apparent digestibility in laying hens.

Items	GEE, mg kg^−1^				*p*-Value	*p*-Value	
	CON	200	400	600		Linear	Quadratic
DM, %	74.07 ± 0.29	70.05 ± 0.09	72.31 ± 0.78	70.21 ± 0.46	0.356	0.054	0.065
CP, %	52.28 ± 3.88	52.44 ± 2.02	52.87 ± 2.2	53.15 ± 4.07	0.986	0.689	0.926
EE, %	81.69 ± 3.11^b^	86.08 ± 0.49^a^	87.2 ± 0.28^a^	87.44 ± 1.21^a^	0.010	0.004	0.003
Ca, %	38.78 ± 2.49	41.6 ± 0.59	42.59 ± 1.78	42.92 ± 6.63	0.531	0.151	0.312
P, %	32.58 ± 2.35	36.35 ± 1.5	34.15 ± 4.47	30.07 ± 3.32	0.171	0.315	0.080

DW, dry matter; CP, crude protein; EE, crude fat; Ca, calcium; P, phosphorus.

^a, b^Means in the same row with different superscripts differ significantly (*p* < 0.05).

### 3.3 Egg quality parameters of laying hens among different GEE treatments

Egg quality parameters of laying hens are presented in Table 4. The addition of GEE had no significant effect on the eggshell thickness, albumen height, Haugh unit, yolk color, or egg shape index during the overall trial.

**Table 4:** Effects of ginger ethanol extract on the egg quality in laying hens.

Items	GEE, mg kg^−1^				*p*-Value	*p*-Value	
	CON	200	400	600		Linear	Quadratic
Egg shell thickness (ESS), mm							
47 weeks	0.38 ± 0.01	0.38 ± 0.01	0.38 ± 0.02	0.37 ± 0.01	0.416	0.466	0.341
51 weeks	0.37 ± 0.01	0.37 ± 0.02	0.37 ± 0.01	0.37 ± 0.01	0.780	0.411	0.595
Albumen height, mm							
47 weeks	6.37 ± 0.25	6.13 ± 0.28	6.05 ± 0.18	6.05 ± 0.41	0.229	0.060	0.110
51 weeks	4.23 ± 0.05	4.15 ± 0.22	4.12 ± 0.46	4.13 ± 0.05	0.863	0.465	0.684
Haugh units							
47 weeks	77.25 ± 2.44	78.72 ± 2.83	79.30 ± 1.11	78.42 ± 2.82	0.523	0.356	0.319
51 weeks	60.97 ± 2.92	60.37 ± 2.23	60.77 ± 6.72	60.48 ± 1.53	0.993	0.879	0.984
Yolk color							
47 weeks	13.70 ± 0.06	13.70 ± 0.19	13.73 ± 0.15	13.83 ± 0.08	0.278	0.078	0.140
51 weeks	13.57 ± 0.1	13.55 ± 0.05	13.60 ± 0.09	13.62 ± 0.18	0.753	0.343	0.606
Egg shape index, mm							
47 weeks	1.31 ± 0.01	1.31 ± 0.01	1.31 ± 0.01	1.31 ± 0.02	0.182	0.673	0.397
51 weeks	1.30 ± 0.02	1.31 ± 0.01	1.31 ± 0.02	1.32 ± 0.01	0.166	0.029	0.082

Different lowercase letters of peer shoulder notes indicate significant differences (*p* < 0.05), and no letters or identical letters indicate no significant difference (*p* > 0.05).

### 3.4 Serum biochemical parameters of laying hens among different GEE treatments

Serum biochemical parameters of laying hens are shown in Table 5. The results demonstrated that the addition of 600 mg kg^−1^ GEE significantly (*p* < 0.05) decreased TG and LDL-C levels compared to the control group. Similarly, the addition of GEE significantly increased TP and HDL-C (*p* < 0.05). Laying hens supplemented with 200 mg kg^−1^ GEE exhibited a higher value for ALB (*p* < 0.05) than the control group, whereas no significant difference was observed among the 400 and 600 mg kg^−1^ GEE groups. GEE supplementation had no significant effect on ALT, AST, and TC levels.

**Table 5:** Effects of ginger ethanol extract on serum biochemical indexes in laying hens.

Items	GEE, mg kg^−1^				*p*-Value	*p*-Value	
	CON	200	400	600		Linear	Quadratic
ALT, U L^−1^	62.13 ± 6.90	53.93 ± 16.04	53.23 ± 13.53	51.63 ± 15.11	0.777	0.399	0.461
AST, U L^−1^	222.08 ± 11.23	229.72 ± 5.57	230.34 ± 7.05	224.73 ± 3.91	0.272	0.060	0.100
ALB, g L^−1^	19.25 ± 0.63^b^	23.68 ± 2.80^a^	20.30 ± 1.27^b^	21.12 ± 1.75^b^	0.003	0.405	0.065
TP, g L^−1^	37.88 ± 2.71^b^	45.80 ± 5.46^a^	50.27 ± 5.53^a^	46.25 ± 6.21^a^	0.004	0.006	0.020
TG, mmol L^−1^	13.10 ± 0.97^a^	12.91 ± 1.11^ab^	11.38 ± 1.09^b^	8.83 ± 0.91^c^	<0.001	0.061	0.196
TC, mmol L^−1^	2.47 ± 0.29	2.30 ± 0.55	2.15 ± 0.04	2.17 ± 0.06	0.556	0.876	0.086
LDL-C, g L^−1^	2.18 ± 0.14^a^	2.12 ± 0.04^a^	1.66 ± 0.09^bc^	1.56 ± 0.06^c^	<0.001	0.268	0.165
HDL-C, g L^−1^	0.36 ± 0.01^b^	0.48 ± 0.03^a^	0.53 ± 0.06^a^	0.41 ± 0.15^ab^	0.004	0.196	0.171

AST, aspartate aminotransferase; ALT, alanine aminotransferase; GLU, glucose; ALB, albumin; TP, total protein; TG, total triglyceride; TC, total cholesterol; LDL-C, low-density lipoprotein cholesterol; HDL-C, high-density lipoprotein cholesterol. ^a, b, c^ Means in the same row with different superscripts differ significantly (*p* < 0.05).

### 3.5 Serum antioxidant parameters of laying hens among different GEE treatments

The effects of dietary GEE supplementation on serum antioxidant parameters of laying hens are summarized in Table 6. Compared to the control group, GEE supplementation significantly elevated SOD, GSH-Px, CAT, and T-AOC levels (*p* < 0.05), with a quadratic dose response (*p* < 0.001). Conversely, MDA levels were significantly reduced in the GEE-supplemented groups (*p* < 0.05), with a quadratic dose response (*p* < 0.001).

**Table 6:** Effects of ginger ethanol extract on serum antioxidant status in laying hens.

Items	GEE, mg kg^−1^				*p*-Value	*p*-Value	
	CON	200	400	600		Linear	Quadratic
SOD, U ml^−1^	3656.65 ± 119.95^c^	4209.72 ± 40.55^b^	4754.66 ± 230.88^a^	4509.73 ± 326.92^ab^	0.001	0.359	<0.001
GSH-Px, U ml^−1^	5636.63 ± 298.95^d^	8895.39 ± 791.64^c^	11181.03 ± 835.93^b^	13436.89 ± 889.04^a^	<0.001	0.031	<0.001
CAT, U ml^−1^	74.62 ± 4.19^d^	93.75 ± 5.48^c^	154.94 ± 11.40^b^	183.17 ± 14.97^a^	<0.001	0.068	<0.001
T-AOC, nmol ml^−1^	2.05 ± 0.59^b^	4.09 ± 0.63^a^	4.09 ± 0.66^a^	4.02 ± 0.89^a^	<0.001	0.831	<0.001
MDA, mmol ml^−1^	94.48 ± 3.27^a^	47.17 ± 4.96^b^	46.71 ± 1.93^b^	44.95 ± 1.93^b^	<0.001	0.507	<0.001

SOD, superoxide dismutase; GSH-Px, glutathione peroxidase; CAT, catalase; T-AOC, total antioxidant capacity; MDA, malondialdehyde. ^a, b, c^ Means in the same row with different superscripts differ significantly (*p* < 0.05).

### 3.6 Serum reproductive hormones of laying hens among different GEE treatments

The effect of GEE supplementation on serum reproductive hormone levels in laying hens is presented in Table 7. The results showed that 600 mg kg^−1^ GEE supplementation significantly increased E_2_ levels compared to the control group (*p* < 0.05), whereas no significant differences were observed in the 200 and 400 mg kg^−1^ GEE groups. However, LH, P_4_, and FSH levels remained unaffected by GEE supplementation at any dose.

**Table 7:** Effects of ginger ethanol extract on serum reproductive hormones in laying hens.

Items	GEE, mg kg^−1^				*p*-Value	*p*-Value	
	CON	200	400	600		Linear	Quadratic
E_2_, pmol g^−1^	0.60 ± 0.05^b^	0.64 ± 0.02^ab^	0.66 ± 0.02^ab^	0.69 ± 0.04^a^	0.053	0.004	0.015
LH, ng g^−1^	0.29 ± 0.04	0.30 ± 0.03	0.29 ± 0.03	0.31 ± 0.01	0.579	0.265	0.002
P_4_, pmol g^−1^	15.50 ± 27.69	15.87 ± 0.43	16.84 ± 1.38	16.88 ± 0.53	0. 634	0.194	0.178
FSH, U g^−1^	0.08 ± 0.01	0.08 ± 0.01	0.08 ± 0.01	0.09 ± 0.01	0.473	0.292	0.436

E_2_, estradiol; LH, luteinizing hormone; P_4_, progesterone; FSH, follicle-stimulating hormone. ^a, b, c^ Means in the same row with different superscripts differ significantly (*p* < 0.05).

### 3.7 Summary of sequence analysis

**Figure 1:**
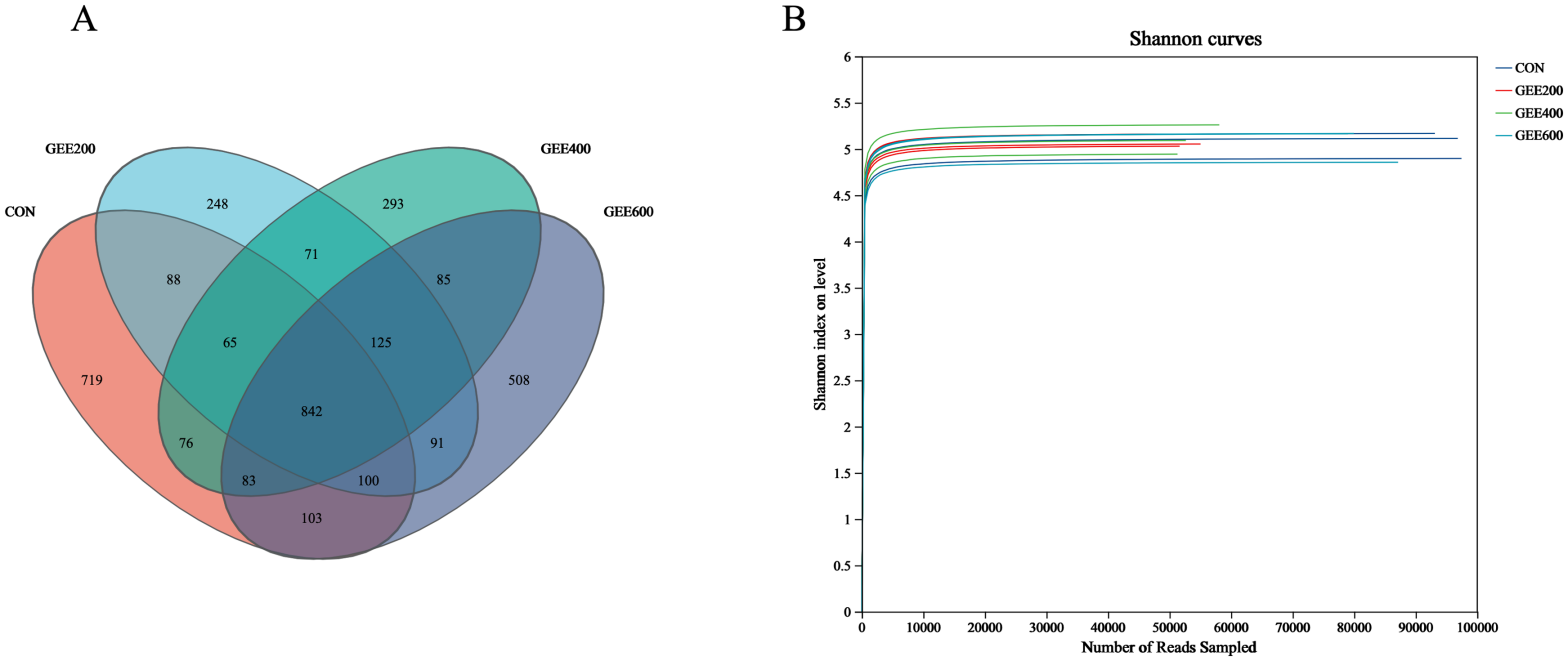
Venn diagram and diversity rarefaction curves of the cecal microbiota. **(A)** Venn diagram of OTU distribution among groups; **(B)** diversity rarefaction curves (Shannon index) verify sequencing adequacy in GEE-fed hens through asymptotic stabilization.

Across all dietary groups, 3,497 OTUs were identified as core microbiota shared among treatment groups. Distinct OTU distributions were observed for each group: CON contained 719 unique OTUs, GEE200 contained 248 OTUs, GEE400 contained 293 OTUs, and GEE600 contained 508 OTUs (Figure 1A). Figure 1B displays rarefaction curves reaching asymptotic saturation, evidencing adequate sequencing depth (saturation plateau attainment) and near-complete taxonomic coverage across samples.

### 3.8 Diversity of the cecal microbiota of laying hens

**Figure 2:**
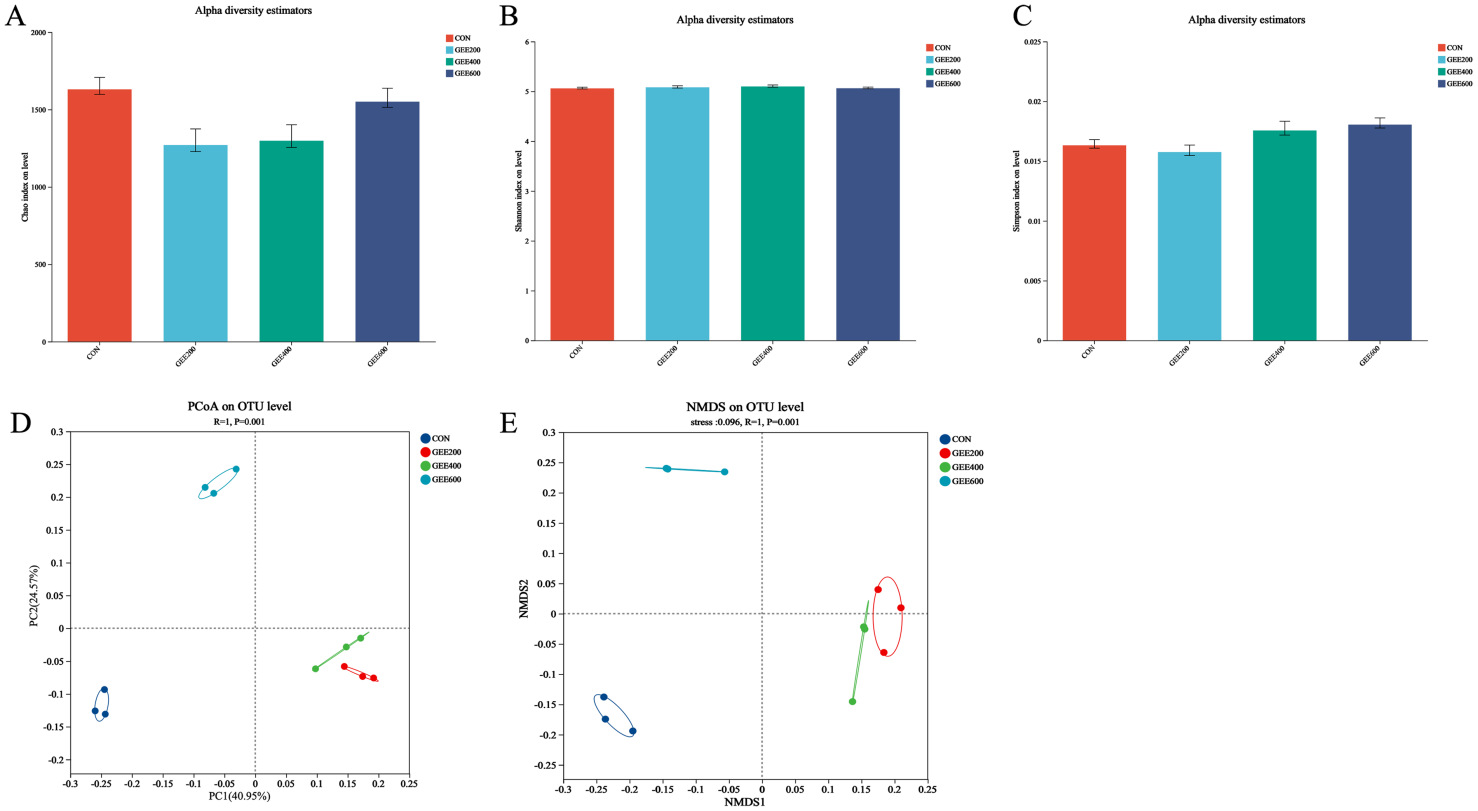
Analysis of cecal microbiota in response to dietary ginger essential oil. The alpha and beta diversity indices of the ceca content in laying hens, assessed by **(A)** Chao index, **(B)** Shannon index, **(C)** Simpson index, **(D)** principal coordinate analysis (PCoA), and **(E)** non-metric multidimensional scaling (NMDS).

Alpha diversity in the ceca of laying hens at day 56 is presented in Figures 2A–E. Compared to the control group, dietary GEE supplementation significantly reduced the Chao indices (*p* < 0.05). The Shannon and Simpson indices were unaffected by the addition of GEE to the diet of laying hens. Multivariate analyses of Bray–Curtis distances using PCoA (axis1:40.94% variance) and NMDS (stress = 0.096) revealed a significant clustering pattern (*R*^2^ = 1.00, *p* = 0.001) between the GEE-treated and control cecal microbiota.

### 3.9 Phylum-level dynamics of laying hens’ cecal microbiota

**Figure 3:**
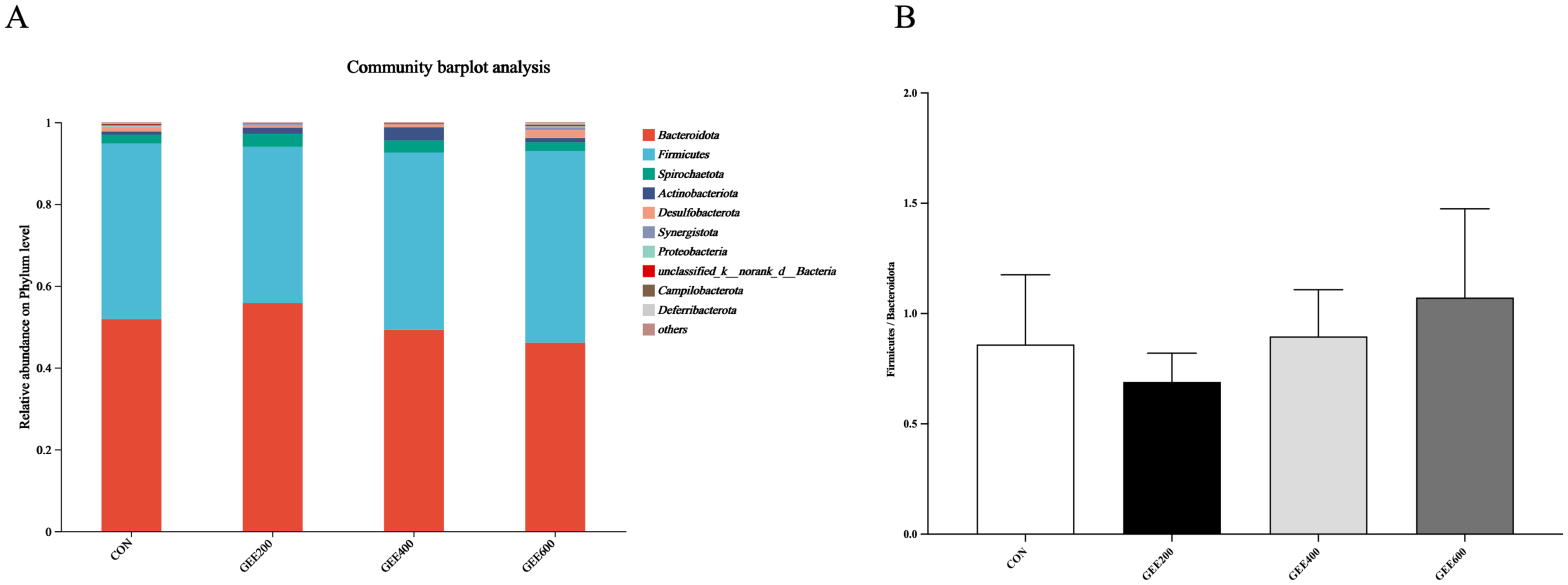
Effects of dietary supplementation with different levels of GEE on the cecal microbiota structure and composition of laying hens at the phylum level. **(A)**The relative abundances of the top 10 cecal microbiota at the phylum level; **(B)** The ratio of Firmicutes/Bacteroidota.

The top 10 dominant bacterial phyla at the phylum level in the ceca microbiota of laying hens, as influenced by dietary supplementation with GEE, are illustrated in Figure 3A. These include *Bacteroidota* and *Firmicutes* among the groups, as well as *Spirochaetota*, *Actinobacteriota*, *Desulfobacterota*, *Synergistota*, *Proteobacteria*, *_unclassified_k__norank_d__Bacteria*, *Campilobacterota*, and *Deferribacterota*, with no significant intergroup differences observed. In addition, the predominant genera included *Bacteroidota* and *Firmicutes* among the groups, accounting for over 90% of the total microbiota in the cecal microbiota of laying hens. Compared to the control group (Figure 3B), the *Firmicutes*/*Bacteroidota* ratio was elevated in the GEE400 and GEE600 groups.

### 3.10 Genus-level dynamics of laying hens’ cecal microbiota

**Figure 4:**
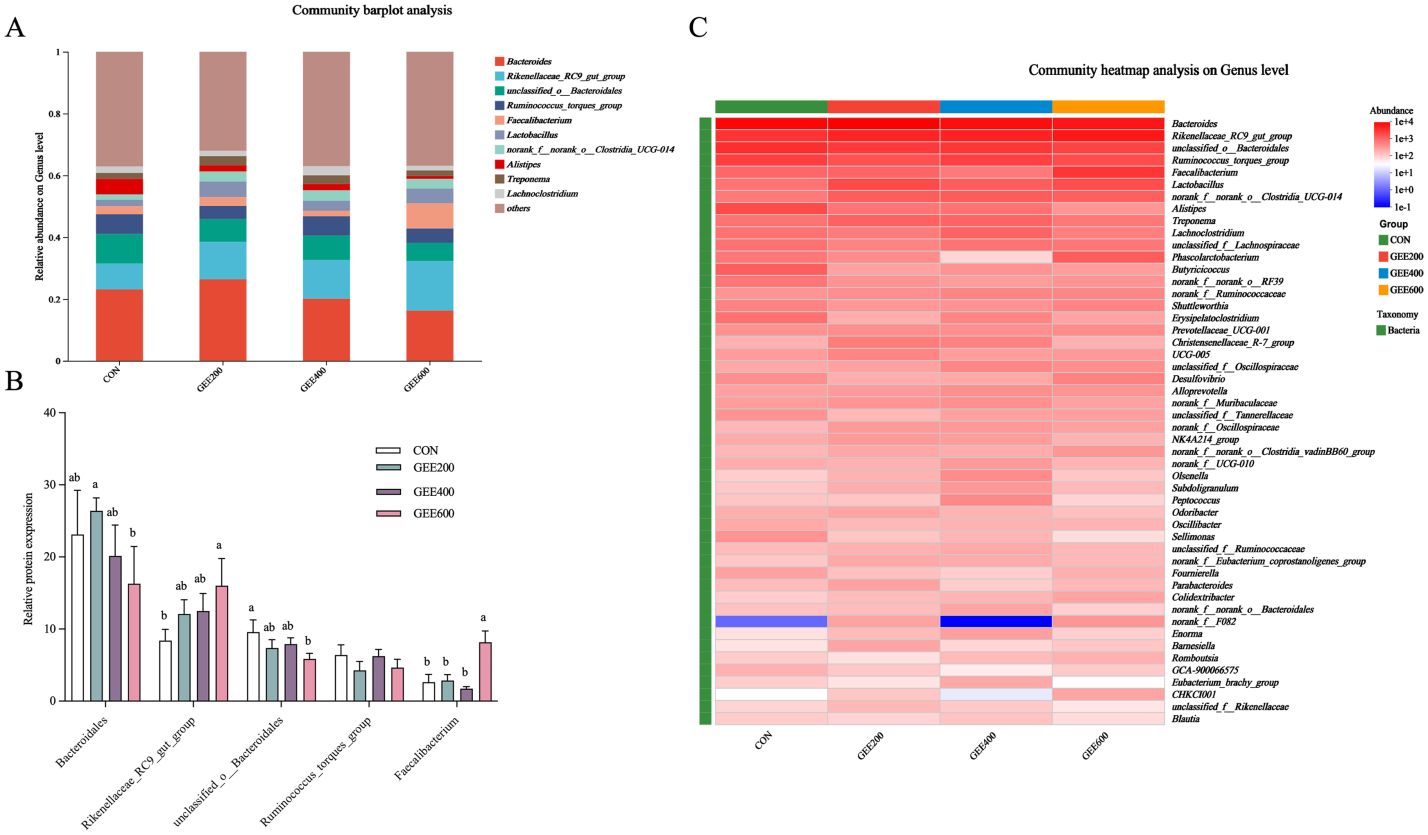
Effects of dietary supplementation with different levels of GEE on the cecal microbiota structure and composition of laying hens at the genus level. The changes in the intestinal microbiota at the genus level **(C–E)**.

The abundance of the top 10 bacterial genera at the genus level in the cecal microbiota of laying hens among different GEE treatments is illustrated in Figures 4A–C. Predominant genera included *Bacteroides*, *Rikenellaceae_RC9_gut_group*, *unclassified_o__Bacteroidalesi, Ruminococcus_torques_group*, and *Faecalibacterium* among groups. Heatmap visualization of species-level abundance is shown in Figure 4A, with the top five dominant genera at the genus level displayed in Figure 4B. Compared to the control group, the 600 mg kg^−1^ GEE group showed significantly increased abundance of *Faecalibacterium* and *Rikenellaceae_RC9_gut_group* (*p* < 0.05), but reduced abundance of *Bacteroides* and *unclassified_o__Bacteroidalesi* in the 600 mg kg^−1^ GEE group were lower (*p* < 0.05). No significant differences were observed between the 200 and 400 mg kg^−1^ GEE groups, and *Ruminococcus_torques_group* abundance remained unaffected across treatments.

### 3.11 Genus LEfSe analysis

**Figure 5:**
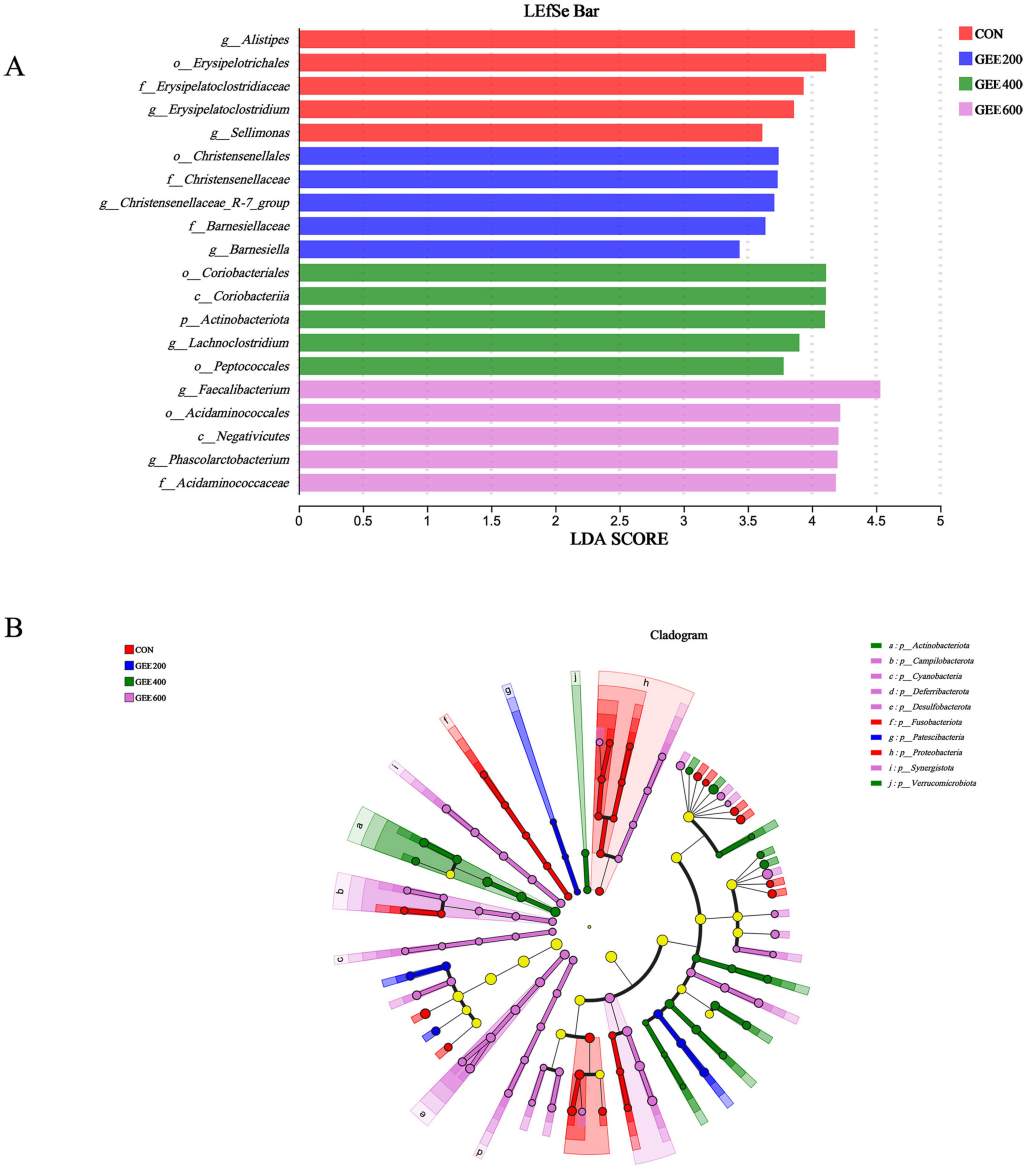
Characterization of the cecal microbiome of laying hens based on linear discriminant analysis effect size (LEfSe) and linear discriminant analysis (LDA) scores. The LEfSe analysis based on order to genus levels (LDA threshold >2.0, *p* < 0.05) **(A)** and a phylogenetic cladogram illustrate their taxonomic hierarchies and evolutionary relationships **(B)**.

Linear discriminant analysis effect size (LEfSe) analysis identified the top five differentially abundant microbial taxa (LDA score >2, *p* < 0.05) in the cecal microbiota of laying hens, with taxonomic distribution visualized in Figure 5A and a phylogenetic cladogram presented in Figure 5B. In the control group, *g__Alistipes*, *o__Erysipelotrichales*, *f__Erysipelatoclostridiacea*, *g__Erysipelatoclostridium,* and *g__Sellimonas* increased. In GEE200, *o__Christensenellales*, *f__Christensenellaceae*, *g__Christensenellaceae_R-7_group*, *f__Barnesiellaceae*, and g*__Barnesiella* markedly increased. In GEE400, *o__Coriobacteriales*, *c__Coriobacteriia*, *p__Actinobacteriota*, *g__Lachnoclostridium*, and *o__Peptococcales* increased. In GEE400, *g__Faecalibacterium*, *o__Acidaminococcale*, *c__Negativicute*, *g__Phascolarctobacterium*, and f*__Acidaminococcaceae* increased.

### 3.12 Correlation analysis

**Figure 6:**
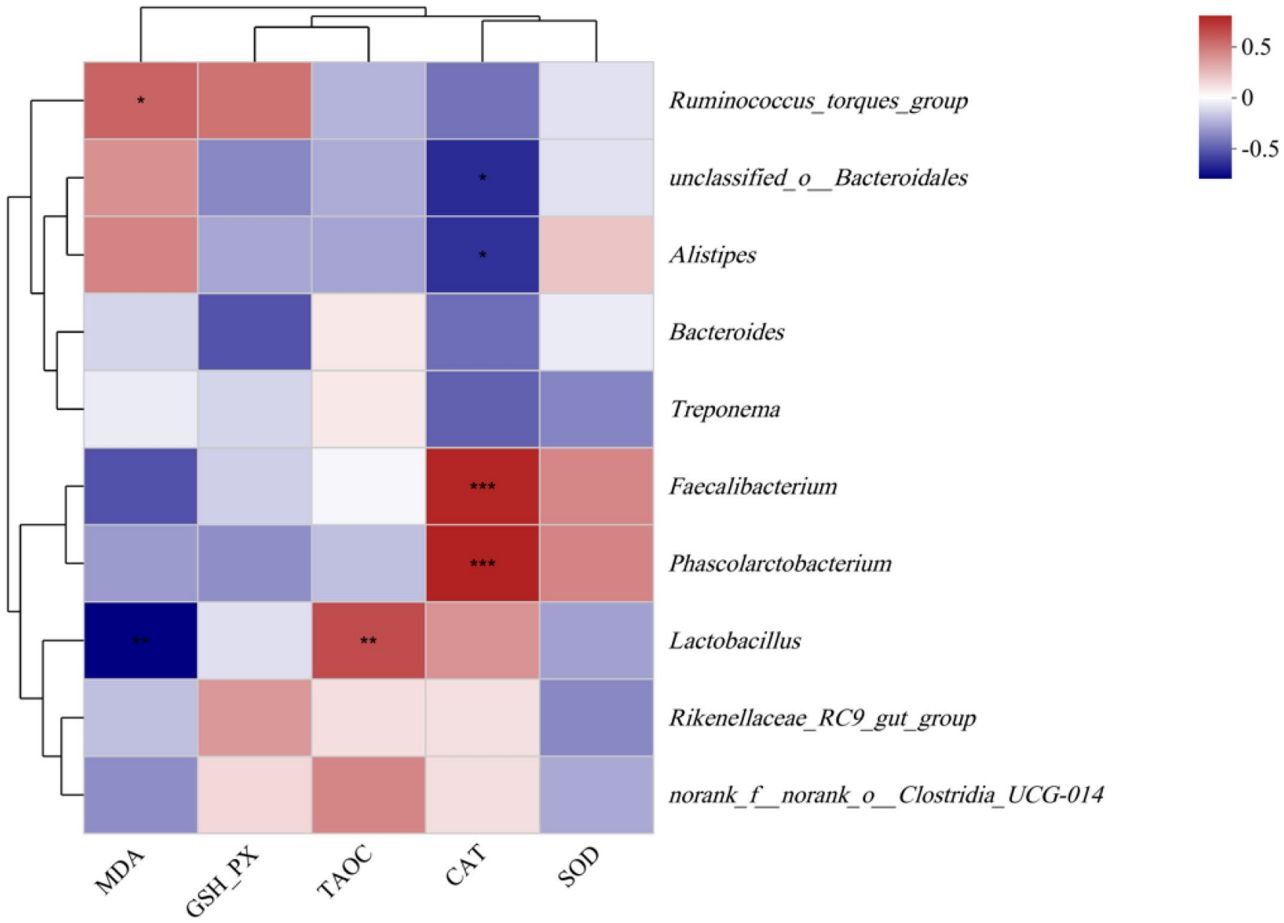
Correlation between the ceca microbial abundances of laying hens and various environmental factors. Colors illustrate correlations, with positive correlations in red and negative correlations in blue. * and ** indicate separately *p* < 0.05 and *p* < 0.01, and *** indicate *p* < 0.001. SOD, superoxide dismutase; GSH-Px, glutathione peroxidase; CAT, catalase; T-AOC, total antioxidant capacity; and MDA, malondialdehyde.

To delineate the functional interplay between gut microbiota dynamics and antioxidant status, Spearman’s rank-order correlation analysis was systematically conducted (Figure 6) to assess associations with serum levels of oxidative biomarkers: malondialdehyde (MDA), glutathione peroxidase (GSH-Px), total antioxidant capacity (T-AOC), catalase (CAT), and superoxide dismutase (SOD) in laying hens. The MDA level was positively correlated with the abundance of *Ruminococcus_torques_group,* whereas the MDA level was negatively correlated with the abundance of *Lactobacillus* (*p* < 0.05). The T-AOC activity was positively correlated with the abundance of *Lactobacillus* (*p* < 0.05). Moreover, the CAT activity was positively correlated with the abundances of *Faecalibacterium* and *Phascolarctobacterium* (*p* < 0.05), whereas the CAT activity was negatively correlated with the abundances of *unclassified_o__Bacteroidales* and *Alistipes* (*p* < 0.05).

### 3.13 Functional prediction based on PICRUSt2

**Figure 7:**
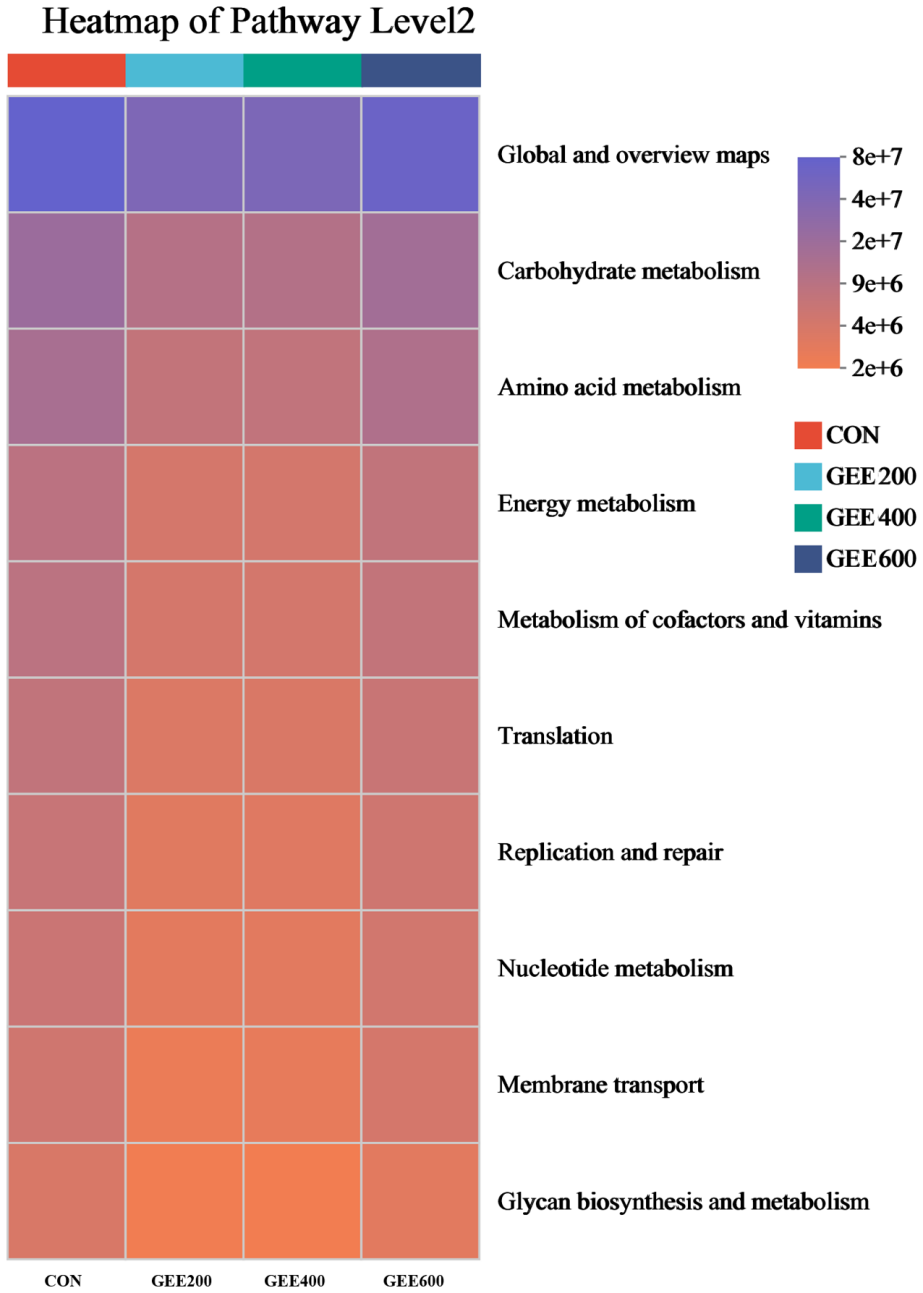
Functional prediction using PICRUSt2.

Following the functional prediction using PICRUSt2 (Figure 7), this study further analyzed the pathway-level functional classifications of the predicted genes. Major enriched pathways included carbohydrate metabolism, amino acid metabolism, energy metabolism, metabolism of cofactors and vitamins, translation, replication and repair, nucleotide metabolism, membrane transport, and glycan biosynthesis and metabolism. These variations in metabolic pathways may influence the antioxidant capacity and performance of laying hens.

## 4 Discussion

Egg-laying hens frequently encounter multiple stressors arising from their environmental conditions and the inherent physiological demands of reproduction (15), which ultimately impair egg production and quality. In recent years, plant extracts have gained attention as sustainable alternatives, offering concentrated sources of key bioactive compounds, including plant essential oils, polyphenols, flavonoids, and polysaccharides. These natural formulations exhibit high potential to enhance egg quality and support digestive function in laying hens (16–18). Ginger oil contains bioactive compounds such as α-zingiberene, β-pinene, camphene, α-farnesene, and β-sesquiphellandrene, which demonstrate strong antioxidant activity, free radical scavenging, metal chelating, and enzyme inhibition capacities (19). In the current study, the dietary supplement GEE did not affect the ADFI, ADEW, or laying rate but significantly increased the feed conversion ratio (FCR) during weeks 5–8 of the experiment. Laying hens fed the 600 mg kg^−1^ GEE-supplemented diet, exhibited the most favorable FCR, while the poorest FCR was recorded in laying hens that were fed the basal diet. The change in FCR due to the supplementation of GEE in the laying hen diet can be attributed to its impact on nutrient digestibility, which is reflected in the improvement of daily body weight gain and FCR (20, 21). The current results of this study are consistent with those of Al-Ghamdi (22), who used a ginger and cinnamon oil mixture (0.25 mL + 0.25 mL kg^−1^ diet) on 1-day-old quail that significantly improved the FCR and antioxidant capacity. In addition, Dosu et al. (23) found that using 0.75% ginger root extract in broiler chickens improved the FCR and body weight. Furthermore, Mohamed et al. (12) reported that dietary supplementation with 500 mg kg^−1^ ginger powder significantly improved the feed conversion ratio for Japanese quail. However, Dosoky et al. (13) demonstrated that ginger powder supplementation (250 and 500 mg kg^−1^) had no significant effect on egg-laying rate, average egg weight, or feed conversion ratio for Japanese quail laying rate from 12 to 21 weeks of age. The discrepancy may be related to the species of avian, the dose of ginger, and the ginger essential oil. Above all, these results demonstrate that dietary GEE or ginger powder is a potent feed additive to increase feed conversion ratio, which may be due to ginger’s ability to improve antioxidant properties in animals. The crude apparent fat (EE) digestibility was significantly improved due to the supplementation of GEE in the diet of laying hens, which explains the improvement of the FCR in laying hens treated with GEE in their diet. Amber et al. (2021) reported that dietary supplementation with ginger root powder in rabbits under heat stress enhanced the growth productivity and nutrient digestibility (24).

Dietary supplementation with GEE had no effect on the eggshell thickness, albumen height, Haugh unit, yolk color, or egg shape index of laying hens among groups, which is inconsistent with the results of Nemati et al. (25), who suggested that supplementation with ginger root powder (0.5, 1, and 1.5 g kg^−1^) has no significant effect on the egg quality of laying Japanese quails, including the shape index, albumen index, albumen, yolk, and shell. The Haugh unit value of eggs from laying hens was evaluated after the administration of GEE in this study, which may be due to the antioxidant properties of ginger essential oil. Previous studies have reported that dietary supplementation with ginger in poultry diets improves the activity of antioxidant enzymes and decreases the content of MDA (26, 27), which benefits egg quality, such as Haugh units and egg yolk (28). Similar to the results in the current study, Wen et al. (9) reported that dietary supplementation with ginger root had no significant effects on shell thickness, albumen height, Haugh unit, or shell strength of laying hens’ eggs, which may be related to the dose of ginger extract. Nemati et al. (25) reported that dietary supplementation with ginger root powder (0.5, 1, and 1.5 g kg^−1^) did not affect the quality of quail eggs (e.g., shape index, albumen index, albumen, yolk, and shell) (25). However, it improved the total antioxidant capacity and decreased the MDA content in serum.

In the present study, the total serum triglycerides and low-density lipoprotein cholesterol decreased with dietary supplementation of GEE in laying hens, while total protein, ALB, and high-density lipoprotein cholesterol increased. These results are consistent with the findings of Herve et al. (29), who reported that laying Japanese quail treated with ginger (*Zingiber officinale, Roscoe*) essential oil at doses of 50, 100, and 150 μL kg^−1^ body weight for 9 weeks showed decreased triglycerides and low-density lipoprotein cholesterol levels. Al-Ghamdi (22) also noted an increase in high-density lipoprotein cholesterol and a decrease in triglycerides and low-density lipoprotein cholesterol in quail treated with ginger and cinnamon oil mixture (0.25 mL + 0.25 mL kg^−1^ diet). Similarly, Habibi et al. (27) demonstrated that dietary supplementation with 150 mg kg^−1^ ginger essential oil significantly increased total protein and albumin levels in the serum of broiler chicks. Qorbanpour et al. (7) reported that broiler chickens receiving 0.25% ginger showed no significant effect on high-density lipoprotein cholesterol and low-density lipoprotein cholesterol concentrations. Lower high-density lipoprotein cholesterol and higher triglyceride levels can result in metabolic syndrome. The results from the current study indicate that dietary supplementation with GEE improved the EE digestibility and the health of laying hens.

Production performance was gradually positively related to the antioxidative capacity of laying hens (30). Shen et al. (31) demonstrated that reactive oxygen species increase during the reproductive process. The activities of total antioxidant capacity, glutathione peroxidase, and superoxide dismutase were lower, and the malondialdehyde level was higher in the livers of 580-day-old laying hens than in those of younger laying hens during the reproductive process and aging. Therefore, improving and repairing the antioxidant capacity are necessary reproductive processes. The antioxidant properties of ginger and GEE have been attributed to the accumulation of antioxidant compounds such as phenolic compounds (32, 33). Ginger (*Zingiber officinale*) has demonstrated antioxidant effects by increasing the activities of glutathione peroxidase and total antioxidant capacity and decreasing the malondialdehyde level (34). Herve et al. (35) reported that dietary supplementation with ginger (*Zingiber officinale, Roscoe*) essential oil improved the antioxidative state of male Japanese quail by increasing the serum antioxidant enzyme activities of catalase, superoxide dismutase, and glutathione, while decreasing the level of malondialdehyde. Notably, the activity of antioxidant enzymes is a crucial indicator of antioxidant function in laying hens. In the present study, the serum contents of superoxide dismutase, glutathione peroxidase, catalase, and total antioxidant capacity were significantly increased, while the serum level of malondialdehyde decreased significantly in laying hens treated with GEE. The increases in superoxide dismutase, glutathione peroxidase, catalase, and total antioxidant capacity, as well as the decrease in malondialdehyde, may be attributed to the antioxidant effect of GEE (35). Overall, these results demonstrate that dietary supplementation with GEE may be a potent antioxidative agent for improving the health status of animals.

The decrease in antioxidant capacity is also associated with changes in the secretion of reproductive hormones, which are closely linked to the production performance of laying hens. He et al. (36) confirmed that the increase in production performance was positively related to the antioxidant capacity of laying hens. El Makawy et al. (37) indicated that the enhancement of antioxidant capacity, including the increase in superoxide dismutase and catalase, and the decrease in malondialdehyde in mice after the addition of ginger oil, was consistent with the elevation of reproductive hormones. This finding aligns with the results of the present research: dietary supplementation with GEE increased the levels of estradiol, luteinizing hormone, progesterone, and follicle-stimulating hormone, further clarifying the reason for the improvement in FCR with the supplementation of GEE in laying hens. Consistent with this study, the increase in production performance and antioxidant capacity is associated with an improvement in reproductive hormones in laying hens (38).

The gut microbiota affects the health, antioxidant capacity, and production performance of animals (11). Therefore, the effects of dietary GEE supplementation on the cecal microbiota in laying hens were sequenced and analyzed. The alpha diversity index reflects the result of the diversity of microorganisms. The higher level of the Chao1 index indicates a lower abundance of dominant species in the community (39). The higher the Shannon index, the greater the community diversity and species distribution, and the higher the Simpson index, the better the uniformity of gut microbiota species (40). In this study, there was no significant effect of dietary supplementation with GEE on the Shannon and Simpson indices. However, a significant decrease in the Chao1 index was observed in the 200 and 400 mg kg^−1^ GEE groups compared to the control and 600 mg kg^−1^ GEE groups, which may be related to the amount of GEE in the diet of laying hens. In addition, previous research demonstrated that a higher FCR is associated with a lower alpha diversity index of the gut microbiota (41, 42), which is consistent with the results of this study.

*Bacteroidota* and *Firmicutes* are key components of the gut microbiota and are known to include short-chain fatty acid-producing bacteria (43), which provide energy for animal metabolic activities by fermenting carbohydrates and degrading plant-derived materials (44). In addition, they promote intestinal barrier function (45), thereby improving the FCR (46). From the phylum level results, it can be depicted that *Bacteroidota* and *Firmicutes* in the ceca of laying hens are predominant, and the *Firmicutes* and *Bacteroidota* ratio increased in the 600 mg kg^−1^ GEE groups compared to the control group. The results of this study were inconsistent with previous research (42, 47), which demonstrated that Firmicutes and Bacteroidota are the predominant phyla in avian microbiota. In addition, *Bacteroidota, Firmicutes,* and *Actinobacteriota* are the dominant gut microbiota, which elevated the FCR in chickens (48). Pretreatment with GEE at levels of 100 and 500 mg kg^−1^ body weight (oral administration) mitigates radiation-induced intestinal oxidative stress in mice (8).

At the genus level, the abundances of *Faecalibacterium* and *Rikenellaceae_RC9_gut_group* significantly increased in the 600 mg kg^−1^ GEE group. *Faecalibacterium* was abundant in the cecal microbiota of laying hens, which is beneficial to humans and animals (49). Wang et al. (50) demonstrated that ginger juice increased the relative abundance of *Faecalibacterium* in the gut microbiota of humans from 5.85 to 7.79%. The increase in *Faecalibacterium* abundance proved that laying hens were in good health after feeding 600 mg kg^−1^ GEE. Health-promoting gut microbes include *Faecalibacterium*, a microbe that produces butyric acid, whose antioxidant effects on the body are fundamentally derived from the regulation of the Nrf2 gene and GSH-Px. The increase in the abundance of *Faecalibacterium* indicated that the level of butyric acid produced increased, thereby increasing its antioxidant capacity (51, 52). Curcumin stimulates the growth of *Lactobacillus* in cecal, and this activity is related to its anti-inflammatory effect. The relative abundance of lactic acid bacteria increased significantly, thereby increasing the potential population of beneficial bacteria in the cecum (53). Luo et al. (54) reported that supplementation with ginger volatile oil increased the abundance of *Lactobacillus* in the ceca of mice, thereby inhibiting the proliferation of MDA-MB-231 breast cancer cells. *Rikenellaceae_RC9_gut_group* in the gut had a potential protective impact by altering the fecal metabolites of weaned piglets (55). All the above results demonstrated that changes in *Faecalibacterium* and *Rikenellaceae_RC9_gut_group* in the ceca are beneficial for animal health, which is inconsistent with this research, as the supplementation of GEE increased the FCR in chickens.

The correlations between antioxidant capacity and gut microbiota showed that bacteria from the genus *Faecalibacterium* had positive associations with antioxidant capacity, while *Ruminococcus_torques_group, unclassified_o__Bacteroidales*, and *Alistipes* had negative relationships with antioxidant capacity. *Faecalibacterium* and *Lactobacillus* have been proven to be beneficial for production performance. According to the functional prediction using PICRUSt2, CUR supplementation may enhance the antioxidant capacity of laying hens by affecting short-chain fatty acid synthesis. The addition of GEE significantly increased their relative abundance at the genus level, indicating that GEE improved antioxidant effects by elevating the relative abundance of intestinal beneficial probiotics.

## 5 Conclusion

In conclusion, GEE increased production performance by improving the FCR, total protein, high-density lipoprotein cholesterol, antioxidant capacity, and estradiol in serum, while decreasing total triglyceride and low-density lipoprotein cholesterol levels in serum, as well as enhancing cecal beneficial microflora in laying hens. Dietary supplementation of 600 mg kg^−1^ GEE to Dawu Golden Phoenix laying hens is the appropriate dosage in the present study. In the future, GEE aligns with global trends toward sustainable and welfare-friendly animal production. The observed improvements in FCR could directly reduce production costs and environmental impact. Moreover, the enhancement of antioxidant capacity suggests the potential for creating premium, value-added products. Based on our results, we propose that a dosage of 600 mg kg^−1^ diet warrants further pilot-scale evaluation as a potential strategy to enhance productivity and sustainability in the laying hen industry. Furthermore, using omics technologies (e.g., RNA-sequencing and metabolomics) would provide a system-level understanding of GEE’s mechanism of action, identifying key genes and metabolic pathways beyond antioxidant responses.

## Data Availability

The raw data supporting the conclusions of this article will be made available by the authors, without undue reservation.

## Footnotes

^1^ https://kns.cnki.net/kcms2/article/abstract?v=VgZR0u5wRbyKtDiN5Uobg6pu30Pg3_sq5Ot5XudaGsqMLSZxWU4sZFBcT9l0m9eTyiMjcnCNPbLjcJFvg21625jlS_EB-isXiuayEpM5ySdHhGjUI7YTY58qyz1HGIAeuIloaiHcXRTp-HhLYTsk5EE_cLwsIjKuDoRtN5RpYP3BDa7cEYWaGqqA==&uniplatform=NZKPT&language=CHS

## References

[1] El-Sabrout K Khalifah A Mishra B. Application of botanical products as nutraceutical feed additives for improving poultry health and production. Vet World. (2023) 16:369–79. doi: 10.14202/vetworld.2023.369-379, PMID: 37041996 PMC10082723

[2] Mosayyeb Zadeh A Mirghelenj SA Hasanlou P Shakouri Alishah H. Effects of pennyroyal (*Mentha Pulegium* L.) supplementation on production performance, egg quality traits, and biochemical parameters of blood and egg in laying hens at later stages of the production period. Vet Med Sci. (2023) 9:242–51. doi: 10.1002/vms3.1031, PMID: 36495177 PMC9857014

[3] Liu B Ma R Yang Q Yang Y Fang Y Sun Z. Effects of traditional Chinese herbal feed additive on production performance, egg quality, antioxidant capacity, immunity and intestinal health of laying hens. Animals (Basel). (2023) 13:2510. doi: 10.3390/ani13152510, PMID: 37570319 PMC10417022

[4] Ding X Cai C Jia R Bai S Zeng Q Mao X. Dietary resveratrol improved production performance, egg quality, and intestinal health of laying hens under oxidative stress. Poult Sci. (2022) 101:101886. doi: 10.1016/j.psj.2022.101886, PMID: 35526444 PMC9092510

[5] Al-Harrasi A Bhtaia S Al-Azri MS Makeen HA Albratty M Alhazmi HA. Development and characterization of chitosan and Porphyran based composite edible films containing ginger essential oil. Polymers (Basel). (2022) 14:1782. doi: 10.3390/polym14091782, PMID: 35566950 PMC9103980

[6] Dos Santos EAR Tadielo LE Schmiedt JA Possebon FS Pereira MO Pereira JG. Effect of ginger essential oil and 6-gingerol on a multispecies biofilm of *Listeria monocytogenes*, *salmonella typhimurium*, and *Pseudomonas aeruginosa*. Braz J Microbiol. (2023) 54:3041–9. doi: 10.1007/s42770-023-01075-2, PMID: 37668830 PMC10689688

[7] Qorbanpour M Fahim T Javandel F Nosrati M Paz E Seidavi A. Effect of dietary ginger (*Zingiber officinale* roscoe) and multi-strain probiotic on growth and carcass traits, blood biochemistry, immune responses and intestinal microflora in broiler chickens. Animals (Basel). (2018) 8:117. doi: 10.3390/ani8070117, PMID: 30011890 PMC6071000

[8] Jeena K Liju VB Ramanath V Kuttan R. Protection against whole body gamma-irradiation induced oxidative stress and Clastogenic damage in mice by ginger essential oil. Asian Pac J Cancer Prev. (2016) 17:1325–32. doi: 10.7314/apjcp.2016.17.3.1325, PMID: 27039766

[9] Wen C Gu Y Tao Z Cheng Z Wang T Zhou Y. Effects of ginger extract on laying performance, egg quality, and antioxidant status of laying hens. Animals (Basel). (2019) 9:857. doi: 10.3390/ani9110857, PMID: 31652863 PMC6912797

[10] Liu J Jin Y Yang J. Influence of spent ginger yeast cultures on the production performance, egg quality, serum composition, and intestinal microbiota of laying hens. Anim Biosci. (2022) 35:1205–14. doi: 10.5713/ab.21.0514, PMID: 35240028 PMC9262721

[11] Li X Sun R Liu Q Gong Y Ou Y Qi Q. Effects of dietary supplementation with dandelion tannins or soybean isoflavones on growth performance, antioxidant function, intestinal morphology, and microbiota composition in Wenchang chickens. Front Vet Sci. (2022) 9:1073659 20230104. doi: 10.3389/fvets.2022.1073659, PMID: 36686185 PMC9846561

[12] Mohamed LA Dosoky WM Kamal M Alshehry G Algarni EH Aldekhail NM. Growth performance, carcass traits and meat physical characteristics of growing Japanese quail fed ginger powder and frankincense oil as feed additives. Poult Sci. (2024) 103:103771. doi: 10.1016/j.psj.2024.103771, PMID: 38749109 PMC11112370

[13] Dosoky WM Farag SA Almasmoum HA Khisheerah NSM Youssef IM Ashour EA. Influences of dietary supplementation of ginger powder and frankincense oil on productive performance, blood biochemical parameters, oxidative status and tissues Histomorphology of laying Japanese quail. Poult Sci. (2023) 102:102988. doi: 10.1016/j.psj.2023.102988, PMID: 37634332 PMC10474497

[14] Nutrition NRCSoP. Nutrient requirements of poultry. Washington: National Academy of Sciences (1950).

[15] Bai K Hao E Huang CX Yue QX Wang DH Shi L. Melatonin alleviates ovarian function damage and oxidative stress induced by dexamethasone in the laying hens through Foxo1 signaling pathway. Poult Sci. (2023) 102:102745. doi: 10.1016/j.psj.2023.102745, PMID: 37302326 PMC10276286

[16] Iqbal Y Cottrell JJ Suleria HAR Dunshea FR. Gut microbiota-polyphenol interactions in chicken: a review. Animals (Basel). (2020) 10:1391. doi: 10.3390/ani10081391, PMID: 32796556 PMC7460082

[17] Wang C Liu X Sun X Li Y Yang X Liu Y. Dietary betaine supplementation improved egg quality and gut microbes of laying hens under dexamethasone-induced oxidative stress. Poult Sci. (2024) 103:104178. doi: 10.1016/j.psj.2024.104178, PMID: 39154612 PMC11381779

[18] Patterson PH Acar N Ferguson AD Trimble LD Sciubba HB Koutsos EA. The impact of dietary black soldier Fly larvae oil and meal on laying hen performance and egg quality. Poult Sci. (2021) 100:101272. doi: 10.1016/j.psj.2021.101272, PMID: 34237547 PMC8267591

[19] Höferl M Stoilova I Wanner J Schmidt E Jirovetz L Trifonova D. Composition and comprehensive antioxidant activity of ginger (*Zingiber officinale*) essential oil from Ecuador. Nat Prod Commun. (2015) 10:1934578X1501000672. doi: 10.1177/1934578X1501000672, PMID: 26197557

[20] Isabel B Santos Y. Effects of dietary organic acids and essential oils on growth performance and carcass characteristics of broiler chickens. J Appl Poult Res. (2009) 18:472–6. doi: 10.3382/japr.2008-00096

[21] Ocak N Erener G Burak Ak F Sungu M Altop A Ozmen A. Performance of broilers fed diets supplemented with dry peppermint (Mentha Piperita L.) or thyme (*Thymus Vulgaris* L.) leaves as growth promoter source. Czeh J Anim Sci. (2008) 53:169–75. doi: 10.17221/373-cjas

[22] Al-Ghamdi ES. Use of ginger and cinnamon oils mixture as a natural alternative to antibiotics in quail feed. Rend Lincei Sci Fis Nat. (2022) 33:843–9. doi: 10.1007/s12210-022-01106-4

[23] Dosu G Obanla TO Zhang S Sang S Adetunji AO Fahrenholz AC. Supplementation of ginger root extract into broiler chicken diet: effects on growth performance and immunocompetence. Poult Sci. (2023) 102:102897. doi: 10.1016/j.psj.2023.102897, PMID: 37562125 PMC10432838

[24] Amber K Badawy NA El-Sayd ENA Morsy WA Dawood MAO. Ginger Root Powder Enhanced the Growth Productivity, Digestibility, and Antioxidative Capacity to Cope with the Impacts of Heat Stress in Rabbits. Journal of Thermal Biology. (2021) 100:103075. 34503812 10.1016/j.jtherbio.2021.103075

[25] Nemati Z Moradi Z Alirezalu K Besharati M Raposo A. Impact of ginger root powder dietary supplement on productive performance, egg quality, antioxidant status and blood parameters in laying Japanese quails. Int J Environ Res Public Health. (2021) 18:2995. doi: 10.3390/ijerph18062995, PMID: 33803951 PMC8001588

[26] Zhang GF Yang ZB Wang Y Yang WR Jiang SZ Gai GS. Effects of ginger root (*Zingiber Officinale*) processed to different particle sizes on growth performance, antioxidant status, and serum metabolites of broiler chickens. Poult Sci. (2009) 88:2159–66. doi: 10.3382/ps.2009-00165, PMID: 19762870

[27] Habibi R Sadeghi G Karimi A. Effect of different concentrations of ginger root powder and its essential oil on growth performance, serum metabolites and antioxidant status in broiler chicks under heat stress. Br Poult Sci. (2014) 55:228–37. doi: 10.1080/00071668.2014.887830, PMID: 24697550

[28] Akbarian A Golian A Sheikh Ahmadi A Moravej H. Effects of ginger root (*Zingiber officinale*) on egg yolk cholesterol, antioxidant status and performance of laying hens. J Appl Anim Res. (2011) 39:19–21. doi: 10.1080/09712119.2011.558612

[29] Herve T Raphael KJ Ferdinand N Victor Herman N Willy Marvel NM Cyril D'Alex T. Effects of ginger (*Zingiber Officinale*, roscoe) essential oil on growth and laying performances, serum metabolites, and egg yolk antioxidant and cholesterol status in laying Japanese quail. J Vet Med. (2019) 2019:7857504. doi: 10.1155/2019/7857504, PMID: 31001562 PMC6436365

[30] Liu X Lin X Zhang S Guo C Li J Mi Y. Lycopene ameliorates oxidative stress in the aging chicken ovary via activation of Nrf2/ho-1 pathway. Aging (Albany NY). (2018) 10:2016–36. doi: 10.18632/aging.101526, PMID: 30115814 PMC6128425

[31] Shen M Jiang Y Guan Z Cao Y Li L Liu H. Protective mechanism of FSH against oxidative damage in mouse ovarian granulosa cells by repressing autophagy. Autophagy. (2017) 13:1364–85. doi: 10.1080/15548627.2017.1327941, PMID: 28598230 PMC5584866

[32] He J Hadidi M Yang S Khan MR Zhang W Cong X. Natural food preservation with ginger essential oil: biological properties and delivery systems. Food Res Int. (2023) 173:113221. doi: 10.1016/j.foodres.2023.113221, PMID: 37803539

[33] Novakovic S Jakovljevic V Jovic N Andric K Milinkovic M Anicic T. Exploring the antioxidative effects of ginger and cinnamon: a comprehensive review of evidence and molecular mechanisms involved in polycystic ovary syndrome (Pcos) and other oxidative stress-related disorders. Antioxidants (Basel). (2024) 13:392. doi: 10.3390/antiox13040392, PMID: 38671840 PMC11047656

[34] Morvaridzadeh M Sadeghi E Agah S Fazelian S Rahimlou M Kern FG. Effect of ginger (*Zingiber Officinale*) supplementation on oxidative stress parameters: a systematic review and Meta-analysis. J Food Biochem. (2021) 45:e13612. doi: 10.1111/jfbc.13612, PMID: 33458848

[35] Herve T Raphael KJ Ferdinand N Laurine Vitrice FT Gaye A Outman MM. Growth performance, serum biochemical profile, oxidative status, and fertility traits in male Japanese quail fed on ginger (*Zingiber Officinale*, roscoe) essential oil. Vet Med Int. (2018) 2018:8. doi: 10.1155/2018/7682060, PMID: 30050674 PMC6046138

[36] He W Wang H Tang C Zhao Q Zhang J. Dietary supplementation with astaxanthin alleviates ovarian aging in aged laying hens by enhancing antioxidant capacity and increasing reproductive hormones. Poult Sci. (2023) 102:102258 20221028. doi: 10.1016/j.psj.2022.102258, PMID: 36435161 PMC9700305

[37] El Makawy AI Ibrahim FM Mabrouk DM Ahmed KA Fawzy Ramadan M. Effect of antiepileptic drug (topiramate) and cold pressed ginger oil on testicular genes expression, sexual hormones and histopathological alterations in mice. Biomed Pharmacother. (2019) 110:409–19. doi: 10.1016/j.biopha.2018.11.146, PMID: 30530043

[38] Liu J Fu Y Zhou S Zhao P Zhao J Yang Q. Comparison of the effect of quercetin and Daidzein on production performance, anti-oxidation, hormones, and Cecal microflora in laying hens during the late laying period. Poult Sci. (2023) 102:102674. doi: 10.1016/j.psj.2023.102674, PMID: 37104906 PMC10160590

[39] Zhao X Zhang Y He W Wei Y Han S Xia L. Effects of small peptide supplementation on growth performance, intestinal barrier of laying hens during the brooding and growing periods. Front Immunol. (2022) 13:925256. doi: 10.3389/fimmu.2022.925256, PMID: 35874672 PMC9301363

[40] Liu JP Wang J Zhou SX Huang DC Qi GH Chen GT. Ginger polysaccharides enhance intestinal immunity by modulating gut microbiota in cyclophosphamide-induced immunosuppressed mice. Int J Biol Macromol. (2022) 223:1308–19. doi: 10.1016/j.ijbiomac.2022.11.104, PMID: 36395935

[41] Siegerstetter SC Schmitz-Esser S Magowan E Wetzels SU Zebeli Q Lawlor PG. Intestinal microbiota profiles associated with low and high residual feed intake in chickens across two geographical locations. PLoS One. (2017) 12:e0187766. doi: 10.1371/journal.pone.0187766, PMID: 29141016 PMC5687768

[42] Zhu C Huang K Bai Y Feng X Gong L Wei C. Dietary supplementation with Berberine improves growth performance and modulates the composition and function of Cecal microbiota in yellow-feathered broilers. Poult Sci. (2021) 100:1034–48. doi: 10.1016/j.psj.2020.10.071, PMID: 33518062 PMC7858044

[43] Chen H Dong L Chen X Ding C Hao M Peng X. Anti-aging effect of phlorizin on D-galactose-induced aging in mice through antioxidant and anti-inflammatory activity, prevention of apoptosis, and regulation of the gut microbiota. Exp Gerontol. (2022) 163:111769 20220322. doi: 10.1016/j.exger.2022.111769, PMID: 35337894

[44] Den Besten G Van Eunen K Groen AK Venema K Reijngoud DJ Bakker BM. The role of short-chain fatty acids in the interplay between diet, gut microbiota, and host energy metabolism. J Lipid Res. (2013) 54:2325–40. doi: 10.1194/jlr.R036012, PMID: 23821742 PMC3735932

[45] Elamin EE Masclee AA Dekker J Pieters HJ Jonkers DM. Short-chain fatty acids activate amp-activated protein kinase and ameliorate ethanol-induced intestinal barrier dysfunction in Caco-2 cell monolayers. J Nutr. (2013) 143:1872–81. doi: 10.3945/jn.113.179549, PMID: 24132573

[46] Wang L Wang J Wang P Liu C Li X Chang J. Effect of corn straw treated with *Lactobacillus Plantarum* and cellulase on ruminal fermentation and microbiota of Hu sheep. Fermentation. (2024) 10:402. doi: 10.3390/fermentation10080402

[47] Waite DW Taylor MW. Characterizing the avian gut microbiota: membership, driving influences, and potential function. Front Microbiol. (2014) 5:223. doi: 10.3389/fmicb.2014.00223, PMID: 24904538 PMC4032936

[48] Li A An Z Li C Cui X Li K Zhou H. Salt-contaminated water exposure induces gut microbial Dysbiosis in chickens. Ecotoxicol Environ Saf. (2023) 254:114731. doi: 10.1016/j.ecoenv.2023.114731, PMID: 36905849

[49] Janczyk P Halle B Souffrant WB. Microbial community composition of the crop and ceca contents of laying hens fed diets supplemented with *Chlorella Vulgaris*. Poult Sci. (2009) 88:2324–32. doi: 10.3382/ps.2009-00250, PMID: 19834082

[50] Wang X Zhang D Jiang H Zhang S Pang X Gao S. Gut microbiota variation with short-term intake of ginger juice on human health. Front Microbiol. (2020) 11:576061. doi: 10.3389/fmicb.2020.576061, PMID: 33708178 PMC7940200

[51] Ma X Fan PX Li LS Qiao SY Li DF. Butyrate promotes the recovering of intestinal wound healing through its positive effect on the tight junctions. J Anim Sci. (2012) 90:266. doi: 10.2527/jas.50965 23365351

[52] Moens F De Vuyst L. Inulin-type Fructan degradation capacity of Clostridium cluster iv and Xiva butyrate-producing Colon Bacteria and their associated metabolic outcomes. Benefic Microbes. (2017) 8:473–90. doi: 10.3920/BM2016.0142, PMID: 28548573

[53] Peng M Zhao X Biswas D. Polyphenols and tri-terpenoids from *Olea europaea* L. in alleviation of enteric pathogen infections through limiting bacterial virulence and attenuating inflammation. J Funct Foods. (2017) 36:132–43. doi: 10.1016/j.jff.2017.06.059

[54] Luo L Chen Y Ma Q Huang Y Xu L Shu K. Ginger volatile oil inhibits the growth of Mda-Mb-231 in the bisphenol a environment by altering gut microbial diversity. Heliyon. (2024) 10:e24388. doi: 10.1016/j.heliyon.2024.e24388, PMID: 38298688 PMC10828689

[55] Liang J Kou S Chen C Raza SHA Wang S Ma X. Effects of *Clostridium Butyricum* on growth performance, metabonomics and intestinal microbial differences of weaned piglets. BMC Microbiol. (2021) 21:85. doi: 10.1186/s12866-021-02143-z, PMID: 33752593 PMC7983215

