# Supplementary material for: The influence of dietary supplementation with ginger ethanol extract on laying hens’ production performance, antioxidant capacity, and gut microbiota
Source: Front Vet Sci. 2025 Sep 23;12:1652982. doi: 10.3389/fvets.2025.1652982 (PMC12502258; doi:10.3389/fvets.2025.1652982)
Supplement: Supplementary file 2 [file Table_2.DOCX]

**T****able S2** The quantification data of major compounds in GEE

| Items | 6-gingerol | 8-gingerol | 10-gingerol |
| --- | --- | --- | --- |
| Sample 1 (mg/g) | 10.29 | 1.62 | 1.64 |
| Sample 2 (mg/g) | 9.95 | 1.57 | 1.60 |
| Sample 3 (mg/g) | 9.81 | 1.54 | 1.56 |
